# Nonmotile Single-Cell Migration as a Random Walk in Nonuniformity: The “Extreme Dumping Limit” for Cell-to-Cell Communications

**DOI:** 10.1155/2018/9680713

**Published:** 2018-11-25

**Authors:** Grigorios P. Panotopoulos, Sebastian Aguayo, Ziyad S. Haidar

**Affiliations:** ^1^BioMAT'X, Facultad de Odontología, Universidad de Los Andes, Santiago, Chile; ^2^School of Dentistry, Pontificia Universidad Católica de Chile, Santiago, Chile; ^3^CIIB, Facultad de Medicina, Universidad de Los Andes, Santiago, Chile

## Abstract

In the present work, we model single-cell movement as a random walk in an external potential observed within the extreme dumping limit, which we define herein as the extreme nonuniform behavior observed for cell responses and cell-to-cell communications. Starting from the Newton–Langevin equation of motion, we solve the corresponding Fokker–Planck equation to compute higher moments of the displacement of the cell, and then we build certain quantities that can be measurable experimentally. We show that, each time, the dynamics depend on the external force applied, leading to predictions distinct from the standard results of a free Brownian particle. Our findings demonstrate that cell migration viewed as a stochastic process is still compatible with biological and experimental observations without the need to rely on more complicated or sophisticated models proposed previously in the literature.

## 1. Introduction

To understand many physiological processes in living organisms, such as embryogenesis and wound healing, among others, as well as their malfunctions, e.g., inflammatory diseases, tumor growth, and metastasis, it is fundamental and of great interest to comprehend the process of relocalization of cells, commonly known as cell migration. This term is used to refer to different processes that involve the movement of cells from one location to another. In living animals, embryonic development provides a clear example of importance of accuracy in cell migration, as errors in this process can result in birth defects. It is also known that proper cell migration is necessary for functional immune response and tissue repair in adults. Conversely, failure in cell migration or inappropriate migratory movements may result in life-threatening scenarios, such as autoimmune diseases, defective wound repair, inflammatory diseases, and tumor dissemination, promoting metastatic cancer progression [1–3].

**Figure 1:**
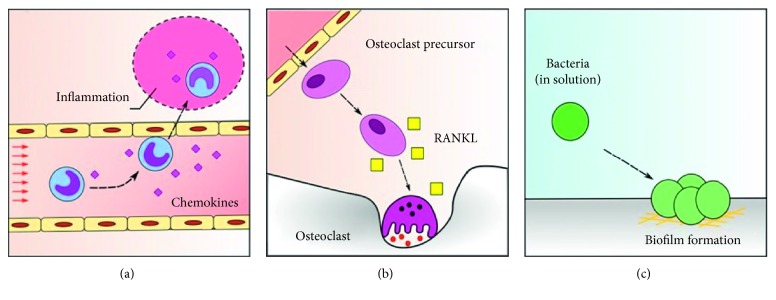
Cell migration is an important factor in physiological and pathological processes. (a) Inflammatory cells can migrate towards a site of interest via the sensing of chemokines and other inflammatory molecules. However, within the blood vessels, they are also subject to other relevant forces such as blood flow. (b) Osteoclast precursor cells are recruited into the tissues, where they can become activated by factors such as receptor activator of nuclear factor kappa-B ligand (RANKL) and cause bone resorption in health and disease. (c) Cell migration is also an important process that allows nonmotile bacteria to attach to surfaces, initiating biofilm formation. Migration of floating bacteria is also determined by important factors such as gravitational forces and flow. In all the above situations, migration is generated by a combination of stochastic (i.e., Brownian motion and random walk) and external forces (i.e., chemokines and flow).

The process of cell migration is very specific and depends on the cell type and the context of the migration process, thus several modes of cell migration have been described [4]. There are migrating cells that are self-propelled (self-driven, with autonomous mobility) and others that are nonmobile. In the case of bacteria, flagella-associated self-propulsion is an important virulence factor for some strains such as *Escherichia coli* and plays a crucial role in attachment to biomaterial surfaces and infection [5, 6]. Additionally, cells can move either as separate entities or by exhibiting a collective behavior. A mathematical model of cell collective movement has been reported [7] as well as a model that explains how swimming velocity of self-driven cells can increase in viscosity [8]. However, in this work we are interested in single-cell movement, which plays a crucial role in maintaining the homeostasis of the body (i.e., leukocyte migration through blood vessels), as well as in tumor progression and metastasis [9]. During these processes, a migrating cell travels through the body by a motion called random walk, yet this process does not fulfill the necessary understanding of the migration process of different cell types. For example, recent evidence has shown that leukocytes exhibit types of migratory behavior which differs from the previously described random walk [10] and that cells can undergo directed migration by the influence of chemical or mechanical signals originating from the environment [11–14] (Figure 1).

At first sight, the migration of nonmotile cells is essentially a random walk, very similar to thermally driven Brownian particles. This is also true for bacteria in suspension. The observation that, when suspended in water, small pollen grains are found to be in a very animated and irregular state of motion was first systematically investigated by Scottish botanist Robert Brown in 1827, and the observed phenomenon took the name of Brownian motion. Albert Einstein in 1905 [15] and sometime later Paul Langevin in 1908 [16] explained Brownian motion using different but equally successful mathematical approaches. Einstein's analysis was based on the diffusion equation:

(1) $$\begin{array}{c} \frac{\partial f\left(t,x\right)}{\partial t}=D\frac{\partial^{2}f\left(t,x\right)}{\partial x^{2}}, \end{array}$$

where *D* is diffusion coefficient, with initial condition *f*(*x*, *t*=0)=*δ*(*x*), where *δ*(*x*) is Dirac's delta function. Solution of diffusion equation is given by [17]

(2) $$\begin{array}{c} f\left(t,x\right)=\frac{1}{\sqrt{4\pi Dt}}\exp\left(-\frac{x^{2}}{4Dt}\right), \end{array}$$

and the mean of the square of displacement is given by 〈*x*^2^〉=2*Dt*. On the other hand, Langevin started from Newton's equation of motion assuming a Stokes's drag force and a random thermal force due to continuous bombardment from molecules of the liquid. Although he did not exploit all richness of his model, Langevin obtained in the long-time regime Einstein's result, namely, by 〈*x*^2^〉=2(*k*_*B*_*T*/6*πaμ*)*t*, where a is the radius of particle, *μ* is fluid viscosity, *T* is temperature, and *k*_B_ is the Boltzmann's constant, hence bridging Brownian motion, random walk, and diffusion, a view soon quantified experimentally by Perrin [18]. Therefore, diffusion coefficient can be computed in terms of properties of the fluid in Brownian particles, which is the Einstein–Stokes formula D  =(*k*_*B*_*T*)/(6*πaμ*).

In modern times, Langevin's approach is still used. In fact, during the last decades, physicist and mathematician modelers have viewed cell movement as a persistent random walk, which can be modeled using well-known stochastic differential equations. The most widely used model is the Ornstein–Uhlenbeck model (OU) [19], with certain predictions that until some years ago were in good agreement with observations and experimental results ([20]). However, recent discoveries seem to question and challenge the archetypical OU model. Specifically, Wua et al., reported that in three dimensions, the path of cells is more directional than random [21]. In addition, recent experiments show a scaling at the long-range regime [22], 〈*x*^2^〉 ~ *t*^*β*^, where the power *β* is just a number in the interval 1 < *β* < 2. It turns out that it is useful to introduce two functions of interest, namely, (i) logarithmic derivative of mean square of displacement (MS) [23], defined previously as

(3) $$\begin{array}{c} \beta \left(t\right)=\frac{d\text{MSD}\left(t\right)}{dt}\frac{t}{\text{MSD}\left(t\right)}, \end{array}$$

and (ii) kurtosis [24]:

(4) $$\begin{array}{c} C\left(t\right)=\frac{\langle {\left(x-\bar{x}\right)}^{4}\rangle }{{\langle {\left(x-\bar{x}\right)}^{2}\rangle }^{2}}, \end{array}$$

where $\bar{x}$ is the mean value of the distribution function. In the special case, where the mean value is zero, kurtosis can be expressed simply, as

(5) $$\begin{array}{c} C\left(t\right)=\frac{\langle x^{4}\rangle }{{\langle x^{2}\rangle }^{2}}, \end{array}$$

since they allow us to make contact between experimental data and predictions of models. Both functions are dimensionless, and in addition, *β(t)* is defined such as in the power law cases, 〈*x*^2^〉 ~ *t*^*β*^, and it is a constant and precisely coincides with the power *β*. In the framework of the OU model, kurtosis is a monotonic function and asymptotically reaches value 3 from below [19], while in [25], data show that kurtosis reaches value 2.3, while in [26] reaches asymptotic value from above. Therefore, in both cases, a departure from standard OU behavior is observed. In light of these findings, the persistent random motion of cell migration has been questioned, and other more complicated theoretical models have been introduced [24, 27] to confront with experimental results.

Therefore, the aim of the present work is to revisit the idea that single-cell motility can be described as a random walk. We point out that previous statements are only valid in case of free Brownian particles. However, if we introduce an external applied force and work in extreme dumping limit, the dynamics change completely, and predictions of the model depend each time on the form of assumed external potential. We define the concept of extreme dumping limit as the extreme nonuniform behavior observed for cell responses and cell-to-cell communications in vivo, within a given biological context. Contrary to previous studies, where authors usually solve the Newton–Langevin equation, here we work with corresponding Fokker–Planck equation and explain why it admits an exact solution for cases we have considered, and we show in plots the kurtosis as well as the logarithmic derivative of mean square of displacement versus time for three different simple models. We show that the nonstandard behavior seen experimentally can be reproduced in the framework of random motion with an applied external force within the extreme dumping limit scenario. Therefore, the random motion paradigm minimally extended can still be used to describe cell motility successfully without the need of more complicated and sophisticated models introduced previously in the literature.

## 2. Results and Discussion

### 2.1. Cell Movement as a Stochastic Process

#### 2.1.1. Newton–Langevin and Fokker–Planck Equations

Let us consider a Brownian particle in one dimension in an external potential *V (x)* with a drag force from the medium proportional to the velocity–*λv* with *λ* given by Stokes' law *λ*=6*πaμ* and a random thermal force (stochastic variable) *ξ*(*t*) that considers the random collisions of the Brownian particle with the molecules of the liquid. This generalizes the OU model where there is no external applied force, and thus describes a free Brownian particle. Newton's equation of motion takes the form

(6) $$\begin{array}{c} ma=f\left(x\right)-\lambda v+\xi \left(t\right), \end{array}$$

where *m* is the mass of the Brownian particle, *f*(*x*)=−*V*′(*x*) is the applied external force, and the random force *ξ*(*t*) is assumed to be a Gaussian white noise:

(7) $$\begin{array}{c} \langle \xi \left(t\right)\rangle =0,\\ \left(\xi \left(t_{1}\right)\xi \left(t_{2}\right)\right)=2g\delta \left(t_{1}-t_{2}\right), \end{array}$$

where *g*=*λkBT* required by the so-called fluctuation-dissipation theorem [28]. In the case of a free particle, there is no external force, *f*(*x*)=0, and we obtain the standard OU model [24]:

(8) $$\begin{array}{c} ma=-\lambda v+\xi \left(t\right), \end{array}$$

and assuming as initial condition *x*0=*x*(0)=0, we obtain for the mean squared of displacement (MSD) (in one dimension), the Furth formula [29]:

(9) $$\begin{array}{c} \langle x^{2}\rangle =2Dt\left[-1+\frac{t}{\tau }+\exp\left(\frac{-t}{\tau }\right)\right], \end{array}$$

where *τ*=*m*/*λ* is the so-called persistent time. In the short-time regime and in the long-time regime, we obtain [30]

(10) $$\begin{array}{c} \langle x^{2}\rangle ∼\left(\frac{k_{B}T}{m}\right)t^{2},\;t⟶0,\\ \langle x^{2}\rangle ∼2Dt,\;t⟶\infty . \end{array}$$

The system eventually exhibits diffusive behavior at late times, while at early times the dynamics are dominated by the inertia of the particle, and the behavior is ballistic, which has been observed in [31].

As it is known that cells are able to feel and sense certain environmental cues such as the stiffness of their environment [32] or surface nanoscale patterning [33], it would be more suitable to extend the standard OU model by considering an applied force that models anything from the external environment that perturbs the movement of the cell, such as signals, stimuli, etc. Furthermore, our focus on extreme dumping limit in which coefficient *λ* in drag force from the fluid is so large that acceleration term *m* a can be neglected. In other words, we can write Newton's equation of motion in the following form:

(11) $$\begin{array}{c} ma+\lambda v=f\left(x\right)+\xi \left(t\right), \end{array}$$

and assume that *λv* ≫ *ma*. In this case the Langevin–Newton equation takes the simpler form:

(12) $$\begin{array}{c} \lambda v≅f\left(x\right)+\xi \left(t\right),\\ \frac{dx}{dt}=A\left(x\right)+\eta \left(t\right), \end{array}$$

where we have defined *A*(*x*)=*f*(*x*)/*λ* and *η*(*t*)=*ξ*(*t*)/*λ*. The latter is the Langevin equation for the process *x*(*t*) where the noise *η*(*t*) satisfies 〈*η*(*t*_1_)*η*(*t*_2_)〉=2*Dδ*(*t*_1_ − *t*_2_) with *D*=*kBT*/*λ*. Since the random force *η*(*t*) is not known, we can only compute mean values of powers of the position, or the moments, 〈*x*^*n*^〉 , once the density probability function is known. The density probability function *u*(*t*, *x*) satisfies the corresponding Fokker–Planck (FP) equation [34]:

(13) $$\begin{array}{c} \frac{\partial u\left(t,x\right)}{\partial t}=D\frac{\partial^{2}u\left(t,x\right)}{\partial x^{2}}-\frac{\partial \left(f\left(x\right)u\left(t,x\right)\right)}{\partial x}, \end{array}$$

where the first term is the diffusion term while the second term is due to the external force with a constant diffusion coefficient *D*. If we ignore the external applied force, the FP equation reduces to standard diffusion equation. That explains why Einstein's approach and Langevin's approach were equally successful. Solving the FP equation, we then can compute the moments performing the integrals:

(14) $$\begin{array}{c} \langle x^{n}\rangle =\int_{-\infty }^{\infty }dw\;\;u\left(t,w\right)w^{n}\;\;, \end{array}$$

and it is a function of time. It is known that this type of Fokker–Planck equation can sometimes be recast in the usual diffusion equation. This happens when the following condition is satisfied [35]:

(15) $$\begin{array}{c} 2DA^{\prime }\left(x\right)+A{\left(x\right)}^{2}=c_{0}+c_{1}x+c_{2}x^{2}, \end{array}$$

and the reason why this happens is that the diffusion equation and the FP equation at hand have the same number of symmetries.

#### 2.1.2. Applications: Three Concrete Simple Models

Next, we shall consider three cases in which we can find exact analytical solution of the FP equation.

- *Constant force or linear potentialV*(*x*)=−*v*_drift_*λx*. A constant force could be for example the gravitational force. In this case the FP takes the form

(16) $$\begin{array}{c} \frac{\partial u\left(t,x\right)}{\partial t}=\;\;\frac{\Delta }{2}\frac{\partial^{2}u\left(t,x\right)}{\partial x^{2}}-v_{\text{drift}}\frac{\partial u\left(t,x\right)}{\partial x}, \end{array}$$

where we have put Δ=2*D*, and it is trivial to check that the condition above is satisfied. So, the FK equation can be recast in the diffusion equation *ω*_*τ*_=*ω*_*yy*_, and the solution is given by *u*(*t*, *x*)=*f*(*t*, *x*)*ω*(*τ*(*t*, *x*), *y*(*t*, *x*)) where *f*(*t*, *x*), *y*(*t*, *x*) and *f*(*t*, *x*) are given by [35]

(17) $$\begin{array}{c} y=x,\\ \tau =\frac{\Delta }{2}t,\\ f\left(t,x\right)=\exp\left(\frac{xv_{\text{drift}}}{\Delta }-\frac{tv_{\text{drift}}2^{}}{2\Delta }\right). \end{array}$$

Therefore, the solution finally is given by [36]

(18) $$\begin{array}{c} u\left(t,x\right)==\frac{1}{\sqrt{2\pi \Delta t}}\exp\left(-\frac{{\left(x-tv_{\text{drift}}\right)}^{2}}{2\Delta t}\right), \end{array}$$

and the mean values 〈*x*^2^〉 and 〈*x*^4^〉 are given by

(19) $$\begin{array}{c} \langle x^{2}\rangle =\Delta t+{\left(tv_{\text{drift}}\right)}^{2},\\ \langle x^{4}\rangle =3{\left(\Delta t\right)}^{2}+{\left(tv_{\text{drift}}\right)}^{4}+6\Delta t^{3}v_{\text{drift}}2^{}, \end{array}$$

where we have made use of the Gaussian integrals:

(20) $$\begin{array}{c} \int_{-\infty }^{\infty }dx\exp\left(-ax^{2}\right)=\frac{\sqrt{\pi }}{\sqrt{a}},\\ \int_{-\infty }^{\infty }dx\;\;x^{2}\exp\left(-ax^{2}\right)=\frac{\sqrt{\pi }}{2a\sqrt{a}},\\ \int_{-\infty }^{\infty }dx\;\;x^{4}\exp\left(-ax^{2}\right)=\frac{3\sqrt{\pi }}{4a{}^{2}\sqrt{a}}. \end{array}$$

It is easy to check that when *v*_drift_ = 0, we recover Einstein's results for the pure diffusion case. Furthermore, it is easy to check that one can obtain the same result by solving the Langevin equation for *x(t)* with the initial condition *x*(0) = 0, namely,

(21) $$\begin{array}{c} x\left(t\right)=tv_{\text{drift}}+\int_{0}^{t}ds\;\;\eta \left(s\right), \end{array}$$

and then by squaring this expression and using the properties of the Gaussian white noise 〈*η*(*t*)〉=0 and 〈*η*(*t*_1_)*η*(*t*_2_)〉=2*Dδ*(*t*_1_ − *t*_2_), one obtains the previous expression for MSD.

Looking at the expression for MSD obtained, the first term is the contribution of the diffusion term, while the second term is the contribution from the applied external force.

Therefore, the system exhibits the diffusive behavior only in the beginning of the evolution, contrary to the OU model, and eventually the deterministic force takes over.

This can explain the observation that sometimes cell motion is more directed than random, such as in the case of cancer cell migration [37] or during zebrafish development [38].

Now the kurtosis *C(t)* as well as the logarithmic derivative of MSD *β*(t) can be computed. We see that for the linear potential case, the kurtosis *C(t)* ⟶ 3 when *t* ⟶ 0 and *C(t)* ⟶ 1 when *t* ⟶ ∞, while the mean squared displacement 〈*x*^2^〉 ~ 2*Dt* at early times (or *β(t)* = 1) and 〈*x*^2^〉 ~ *t*^2^ at late times (or *β(t)* ⟶ 2).

(ii) *Harmonic oscillator or parabolic potentialV*(*x*)=*θλx*^2^/2*that corresponds to a forcef*(*x*)=−*θλx*. It is quite common to model attractive forces with springs ([39, 40]), and thus a harmonic trap is a reasonable potential to consider. In this case, the FP takes the form

(22) $$\begin{array}{c} \frac{\partial u\left(t,x\right)}{\partial t}=D\frac{\partial^{2}u\left(t,x\right)}{\partial x^{2}}+\theta \frac{\partial \left(xu\left(t,x\right)\right)}{\partial x}, \end{array}$$

and the condition above is again satisfied. So, the FK equation can be recast in the diffusion equation *ω*_*τ*_=*ω*_*yy*_, and the solution is given by *u*(*t*, *x*)=*f*(*t*, *x*)*ω*(*τ*(*t*, *x*), *y*(*t*, *x*)), where *f*(*t*, *x*), *y*(*t*, *x*) and *τ*(*t*, *x*) are given by [36]

(23) $$\begin{array}{c} y=x\exp\left(\theta t\right),\\ \tau =\frac{D}{2\theta }\exp\left(2\theta t\right),\\ f\left(t,x\right)=\exp\left(\theta t\right). \end{array}$$

Therefore, the solution finally is given by [34]

(24) $$\begin{array}{c} u\left(t,x\right)=\frac{\sqrt{\theta }}{\sqrt{2\pi D\left(1-\exp\left(-2\theta t\right)\right)}}\exp\left(-\frac{\theta x^{2}}{2D\left(1-\exp\left(-2\theta t\right)\right)}\right), \end{array}$$

and using the same Gaussian integrals as before, the mean values 〈*x*^2^〉 and 〈*x*^4^〉 are given by

(25) $$\begin{array}{c} \langle x^{2}\rangle =\frac{D}{\theta }\left(1-\exp\left(-2\theta t\right)\right),\\ \langle x^{4}\rangle =3{\left(\langle x^{2}\rangle \right)}^{2}, \end{array}$$

and one can check that when *θ*=0, we recover Einstein's results for pure diffusion, and we make use of the fact that

(26) $$\begin{array}{c} \frac{1-\exp\left(-2\theta t\right)}{\theta }⟶2t,\;\theta ⟶0. \end{array}$$

Therefore, the kurtosis in this case is always a constant in time *C(t)* = 3. On the other hand, at early times *t* ⟶ 0 the mean squared 〈*x*^2^〉 ~ 2*Dt* (diffusive behavior or *β*(0) = 1), while at late times *t*⟶*∞*〈*x*^2^〉⟶D/*θ*(or  *β*(*t*)⟶0).

(iii) *Constant force and within a harmonic trapV*(*x*)=*θλx*^2^/2 − *v*_drift_*λx*. This model combines the two previous cases, and by redefinition *z*=*x* − (*v*_drift_/*θ*), we recover the FK equation of the harmonic trap. Therefore, the solution reads

(27) $$\begin{array}{c} u\left(t,x\right)=\frac{\sqrt{\theta }}{\sqrt{2\pi D\left(1-\exp\left(-2\theta t\right)\right)}}\exp\left(-\frac{\theta \;\left(x-{\left(v_{\text{drift}}\left(1-\exp\left(-\theta t\right)\right)/\theta \right)}^{2}\right)}{2D\left(1-\exp\left(-2\theta t\right)\right)}\right). \end{array}$$

Finally, with help of Gaussian integrals and defining

(28) $$\begin{array}{c} X=\frac{v_{\text{drift}}}{\theta }\left(1-\exp\left(-\theta t\right)\right),\\ \alpha =\frac{\theta }{2D\left(1-\exp\left(-2\theta t\right)\right)}, \end{array}$$

the moments are computed to be

(29) $$\begin{array}{c} \langle x^{2}\rangle =X^{2}+\frac{1}{2\alpha },\\ \langle x^{4}\rangle =X^{4}+\frac{3}{4\alpha^{2}}+3\frac{X^{2}}{\alpha }. \end{array}$$

The MSD in short-time regime is diffusive 〈*x*^2^〉 ~ 2*Dt*, while in long-time regime approaches a constant value 〈*x*^2^〉⟶(*D*/*θ*)+(*v*_drift_^2^/*θ*^2^). Kurtosis at early times starts from the value 3, while eventually approaches the value

(30) $$\begin{array}{c} C_{\infty }=\frac{\left(1+6b+3b^{2}\right)}{\left(1+2b+b^{2}\right)}, \end{array}$$

where we have defined dimensionless quantity *b*=*θD*/*v*_drift_^2^. This value depends on the interplay between *θ* and *v*_drift_. The drift velocity (linear term in potential) dominates *b* ≪ 1 and *C*_*∞*_≅1, while *θ* (quadratic term in potential) dominates *b* ≫ 1 and *C*_*∞*_≅3. Therefore, it is possible to reproduce observation of [25] that kurtosis approaches 2.3 value. This can be achieved for *b*≅0.33.

In both the second and third models, MSD asymptotically in time goes to a constant value due to the harmonic trap, and their logarithmic derivatives of MSD exhibit similar behavior, namely, they both are a monotonically decreasing function of time in the interval 0 < *β(t)* < 1.

**Figure 2:**
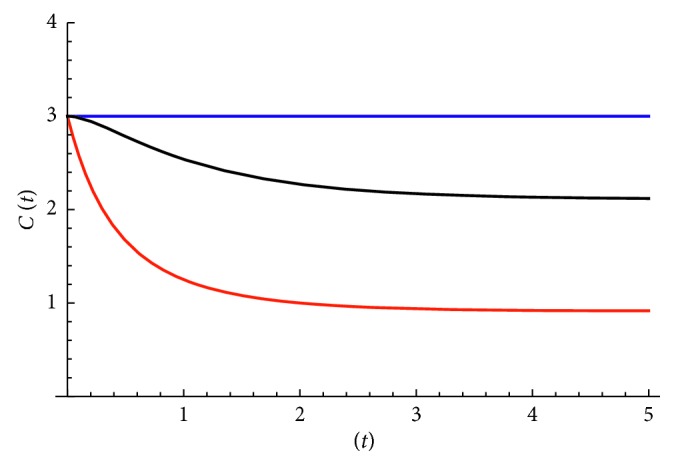
Kurtosis versus time for three models considered in the present work. The constant function corresponds to the parabolic potential (second model, blue color), the one that eventually goes to 1 corresponds to the constant force (first model, red color), and the last one corresponds to the third model that combines the two (constant force in a harmonic trap, black color) for *b* = 0.5. Its asymptotic value depends on the interplay between θ and Vdrift and lies between 1 and 3.

**Figure 3:**
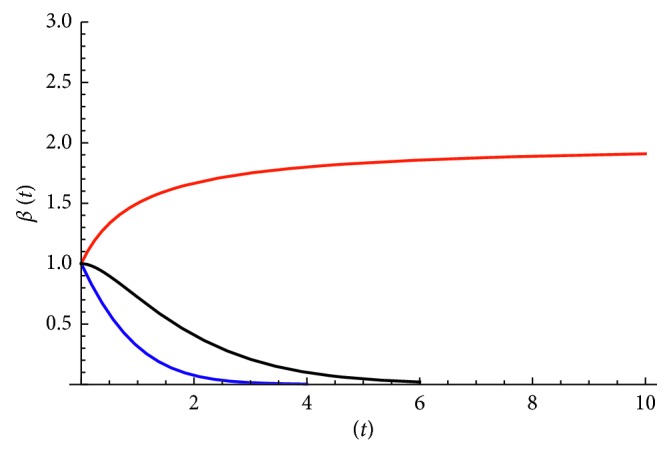
Logarithmic derivative of MSD *β(t)* versus time for the three models considered in the present work. The decreasing function with values in the interval (0, 1] corresponds to the harmonic traps (second model in blue and third model in black), while the increasing function with values in the interval [1, 2) corresponds to the constant force case (first model red color).

In the corresponding Figures, we show functions *C(t)* and *β(t)* versus time for all three models.  Figure 2 shows the kurtosis, while Figure 3 shows *β(t)* for the models considered here.

We see that each model exhibits its own dynamics, and they behave differently at late times, although in the short-range regime (t ⟶ 0), they all exhibit diffusive behavior.

The simplest model with a constant applied force can explain (i) the scaling behavior with power *β* = 2, (ii) the fact that cell movement can be more directed than random, and (iii) the monotonic decrease of kurtosis as seen in [26].

In addition, a more complicated model with a harmonic trap and constant force can explain asymptotic value of 2.3 seen previously [25]. Therefore, our results show that departures from OU model seen in recent experiments are also present here, and thus our findings suggest that the random motion paradigm minimally extended is still compatible with biological observations, at least qualitatively.

It would be interesting to obtain more data that could verify (or falsify) the predictions of the models considered here in a quantitative manner.

#### 2.1.3. Biological Significance

Although it is widely accepted that cell migration is complex and multifactorial, our results show that this process can be described with a modification of the random walk model that also considers the application of external forces within a complex biological environment. Therefore, it remains possible to model cell migration in a minimalistic fashion without the need of using more complex calculations. Furthermore, this model is in line with several experimental observations in the laboratory. For example, despite the fact that fibroblasts can display random migration patterns in 2D tissue culture [20], many groups have observed that fibroblasts are also able to alter their migratory behavior when changed from a 2D to a 3D culture [4, 41]. This change in migration patterns is believed to be due to the ability of cells to detect and respond to stimuli in their environment, such as an increased number of adhesion points or mechanical differences of the matrix. Thus, given the 3D nature of the in vivo setting, it is possible to determine that cell migration within a living organism is more complex than a simple random walk, being also influenced by a number of external cues.

Furthermore, it has also been discussed that macrophages and neutrophils migrate using various modes of random walks (i.e., biased random walks) in response to acute injury, most of which include external factors that are guiding the cell towards an area of interest [42–44]. The presence of these factors (i.e., chemokines) are crucial in the biological setting, as they are responsible for generating effective inflammatory and wound healing responses [45]. Also, chemokine-based migration is imperative for osteoclast recruitment into bone tissue, which plays a big role in bone resorption observed in many chronic inflammatory diseases such as rheumatoid arthritis, periodontitis, and peri-implantitis [46, 47]. In all these scenarios, either constant or complex external forces are at play guiding cell migration towards the area of interest. However, our results strengthen the idea that cell migration is in fact a combination of stochastic processes and directed motion by external stimuli, which is supported by the calculations discussed in this research.

## 3. Conclusion

Studying properties of cell migration is of fundamental interest to understand many physiological processes in living organisms, as well as some pathological processes such as tumor metastasis or bacterial infection. Single-cell motility of nonmotile cells can be viewed as a random walk assuming that cells are thermally driven Brownian particles and can be modeled using well-known stochastic differential equations such as Langevin and Fokker–Planck equations. Recent experimental results have questioned the archetypical Ornstein–Uhlenbeck model, as they have shown some departures from standard predictions of persistent random motion paradigm based on a free Brownian particle driven by a drag Stokes's force, as well as by a random force due to molecule thermal motion. In the present work, we have revisited the issue of cell migration viewed as random walk by adding an applied external force and working in extreme dumping limit. We have studied three concrete cases for which the Fokker–Planck equation can be solved exactly, and we have provided analytical expressions for MSD and for certain quantities of interest that can be used to make contact observations. Our results show that predictions of the model and behavior of the system depend on form of the applied force (although all models exhibit diffusive behavior in short-time regime), and they all differ compared to the standard OU model. Our work shows that random motion paradigm minimally extended can still be used to describe cell motility successfully without introduction of sophisticated models. Overall, this model could be potentially beneficial to understand the migration behavior of cells during relevant biological processes such as wound healing, inflammation, and embryonic development from a minimalistic approach.

## Data Availability

Mathematical calculations utilized to support the findings of this study are included within the article.
